# Correcting intervertebral rotation and scoliosis simultaneously by oblique lumbar interbody fusion: a 3D analysis of EOS images

**DOI:** 10.3389/fsurg.2023.1145059

**Published:** 2023-06-12

**Authors:** Zhongning Xu, Tenghui Ge, Qingyun Li, Ronghui Cai, Jingye Wu, Yuqing Sun

**Affiliations:** Department of Spine Surgery, Peking University Fourth School of Clinical Medicine, Beijing Jishuitan Hospital, Beijing, China

**Keywords:** adult degenerative scoliosis, oblique lateral interbody fusion, EOS imaging system, intervertebral motion, 3D analysis

## Abstract

**Purpose:**

With advancements in minimally invasive techniques, oblique lumbar interbody fusion (OLIF) has gained widespread acceptance and is now commonly performed for adult degenerative scoliosis (ADS). The objective of this research paper is to evaluate three-dimensional (3D) intervertebral motions in EOS models before and after surgery and subsequently assess the efficacy of the 3D correction achieved through staged OLIF.

**Methods:**

In this retrospective study, 29 consecutive patients diagnosed with ADS were included, with a mean age of 63.6 years, who underwent staged OLIF surgery between 2018 and 2021. Spinopelvic parameters were assessed using EOS images, and 3D models were reconstructed to measure intervertebral motion angles (IMAs) in 70 surgical intervertebral segments, comprising wedge, lordosis, and axial rotation angles. Regression analysis was conducted to compare IMAs in different planes before and after the staged OLIF surgery.

**Results:**

Significant three-dimensional correction was observed in 70 intervertebral segments following the first-stage OLIF. The wedge angles decreased from 5.2°± 4.2° to 2.7°± 2.4° (*P* < 0.001). The lordosis angles increased from 5.1°± 5.9° to 7.8°± 4.6° (*P* = 0.014), while the axial rotation angles decreased from 3.8°± 2.6° to 2.3°± 2.1° (*P* < 0.001). Linear regression analysis revealed a positive correlation between wedge angles and axial angles preoperatively (*P* < 0.001, *r* = 0.43), as well as between corrected wedge angles and corrected axial angles (*P* < 0.001, *r* = 0.42).

**Conclusion:**

This study demonstrated that intervertebral motions had a correlation between coronal and axial planes in lumbar degenerative scoliosis. First-stage OLIF was efficient at correcting segmental scoliosis by inserting cages while correcting rotation deformity simultaneously, as well as improving the sagittal spinopelvic parameters.

## 1. Introduction

Adult degenerative scoliosis (ADS), characterized by a Cobb angle ≥10°, has emerged as a significant global public health issue. Patients diagnosed with ADS frequently manifest symptoms such as low back pain, radiculopathy, and neurogenic claudication, which may significantly compromise overall health status and functional ability. Although the precise mechanisms behind ADS remain to be fully elucidated, prevailing theories propose that asymmetric disc degeneration, facet hypertrophy, capsule degeneration, and ligamentous hypertrophy play contributory roles (1). Extant literature has established the involvement of both coronal and sagittal imbalances in the pathogenesis of ADS. However, a comprehensive understanding of the role of axial rotation in the overall deformity remains insufficient (2).

To assess vertebral rotation, anteroposterior plain films are frequently employed in clinical settings. However, this method has been deemed inaccurate because it relies solely on the projection of a single plane, without considering the three-dimensional shape of the vertebrae. In contrast to traditional 2D x-rays, the EOS® images (Paris, France) provide a more extensive evaluation of spinal alignment with ultralow radiation doses (3). In addition, EOS images are captured in weight-bearing positions, which typically coincide with the manifestation of pain and restricted mobility. Subsequently, the sterEOS software reconstructs 3D models of the spine, allowing surgeons to examine intervertebral motion in different planes (4).

Surgical interventions for ADS encompass decompression alone, decompression with limited short fusion, and decompression along with long fusion and (or) correction of deformity (5). Amid advancements in minimally invasive spine surgery, the oblique approach has gained widespread adoption due to minor damage and short operating time (6). Previous reports indicated that ADS can be effectively ameliorated through the insertion of a cage into the intervertebral space, obviating the need for posterior fixation (7). However, there are no clinical studies investigating alterations in intervertebral motions resulting from OLIF.

This study aims to explore the correlations among intervertebral motions using 3D EOS models before and after staged OLIF surgery, and subsequently evaluate the 3D corrective efficacy of the OLIF procedure.

## 2. Materials and methods

### 2.1. Patient selection

The inclusion criteria were as follows: (i) adult patients diagnosed with ADS by clinical symptoms (low back pain, radiculopathy, and neurogenic claudication), along with radiological evidence (Cobb angle ≥10° in X-ray) collected between August 2018 and August 2021; (ii) availability of complete preoperative and postoperative medical records, including EOS images captured before and 1 week after staging OLIF; and (iii) informed consent for the use of anonymized medical data for research purposes. Exclusion criteria were: (i) patients with a history of spinal, pelvic, or lower-extremity surgery; (ii) patients with congenital scoliosis, idiopathic scoliosis, neuromuscular scoliosis, spinal tumors, infections, or traumatic spinal injuries; and (iii) patients with incomplete or missing radiographic imaging data. This retrospective single-center study selected 70 surgical levels in 29 patients.

### 2.2. Surgical procedures

All patients underwent a two-stage OLIF surgery, which was carried out by an experienced spinal surgeon. In the first stage, the patients were placed in the right lateral decubitus position under general anesthesia. Using C-arm guidance, the target segments were identified and a skin incision was made at the anterior of the center of the target segments and parallel to the external oblique muscle. In cases involving two or more levels, the incision was extended to 4.5–6.0 cm. The muscle layers, including the external oblique, the internal oblique, and the transversalis, were bluntly dissected along the direction of the fibers, along with a dissection of retroperitoneal fat tissue. The psoas muscle was retracted posteriorly to expose the intervertebral space. After discectomy, a suitable-sized OLIF cage (Clydesdale Spinal System, Medtronic, Memphis, TN, USA) filled with artificial bone was inserted into the intervertebral space to restore the height of the disc space. It is worth noting that the size of the fusion cage could potentially impact the corrective outcome, and in this study, we selected the size of the fusion cages based on the specific condition of the patients. The employed cages had a lordotic angulation of 6°, a standardized width of 18 mm, and average height and length measurements of 11.7 ± 1.3 mm (8–14 mm) and 53.5 ± 5.2 mm (27–60 mm), respectively. The second-stage surgery was performed approximately 7–10 days after the first-stage surgery, with the patients positioned in the prone position under general anesthesia, and percutaneous pedicle screw fixation was carried out on the target segments.

### 2.3. Imaging study

Standard demographic information was extracted from the patients (age, sex, and body mass index). Biplanar low-dose stereoradiographs were obtained with the patients in a standing position by using the EOS system (EOS Imaging, Paris, France) according to a standardized protocol: free-standing position with a horizontal gaze, with fists on the clavicle to avoid superimposition of the arms with the spine (8). If patients could not hold this position or stand on their own, their hands were placed on the transverse bar as softly as possible. All patients underwent EOS imaging before and approximately 1 week after the first-stage surgery.

### 2.4. 3D modeling

**Figure 1:**
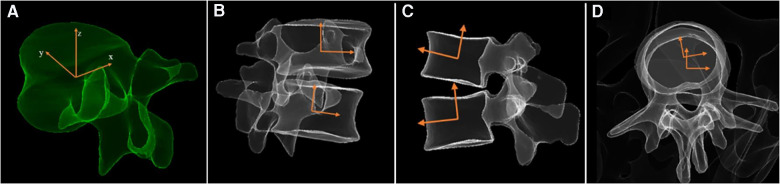
(**A**) 3D Reconstruction and coordinate system of vertebrae. Measurement of intervertebral motion angles in three planes: (**B**) wedge angle, (**C**) lordosis angle, (**D**) axial rotation angle.

An operator used the built-in sterEOS software to reconstruct 3D models on the basis of surgical levels. A cartesian coordinate system was established for each vertebra with the origin defined at the center of the vertebra. As seen in Figure 1, the *X*-axis is parallel to the line connecting the centers of the bilateral pedicles, the *Y*-axis is parallel to the line connecting the midpoint of the bilateral pedicles and the center of the vertebral body, and the *Z*-axis is perpendicular to the plane formed by the *X* and *Y* axes. In this way, the intervertebral motion angles in the three planes could be determined based on the angle between two corresponding axes of adjacent vertebral bodies in the coordinate system.

### 2.5. Spinopelvic parameters

Spinopelvic parameters such as lumbar lordosis (LL), thoracic kyphosis (TK), pelvic incidence (PI), sacral slope (SS), pelvic tilt (PT), mismatch of PI and LL (PI-LL), the sagittal vertical axis (SVA), as well as IMAs were measured using the sterEOS software. As scoliosis orientations vary among patients, it is preferable to use the absolute values for the wedge and axial rotation angles to represent the magnitude of motion, while vector data can be used in correlation analysis. However, there is no need to convert the lordosis angle into an absolute value, as most lumbar vertebrae exhibit lordosis. In this study, all the parameters were measured independently by two spine surgeons to ensure accuracy and consistency.

### 2.6. Statistical analysis

Descriptive analysis included calculating means and standard deviations for all demographic and radiographic parameters. Inter-rater reliability of radiographic parameters was assessed using interclass correlation coefficients (ICCs). A paired *t*-test was performed to compare spinopelvic parameters and all IMAs before and after surgery. Regression analysis was used to evaluate the linear relationships among the IMAs in the three planes, both before and after surgery, as well as their respective difference values. Statistical analysis was conducted using IBM SPSS Statistics v. 26.0.0 (SPSS Inc., 2019, NY, USA). The level of significance was set at *p* < 0.05.

## 3. Result

### 3.1. Patient characteristics

The study included 7 men and 22 women, with a mean age of 63.6 ± 7.1 years and a mean BMI of 26.1 ± 2.92 kg/m^2^ (Table 1).

**Table 1:** Demographic data of patients.

Number of patients (*n*)	29	
Age (year)	63.6 ± 7.1	
Sex (male:female)	7:22	
BMI (kg/m^2^)	26.1 ± 2.9	
Level distribution	L1–L2	5
	L2–L3	15
	L3–L4	22
	L4–L5	28

### 3.2. Three-dimensional intervertebral motions

All three IMAs changed significantly. The wedge angles decreased from 5.2° ± 4.2° to 2.7° ± 2.4°, and the rotation angles decreased from 3.8° ± 2.6° to 2.3° ± 2.1°. Correspondingly, the lordosis angles increased from 5.1° ± 5.9° to 7.8° ± 4.6° (Table 2).

**Table 2:** 3d alignment segmental parameters before and after surgery.

	Pre-op (inter-rater ICC)	Post-op (inter-rater ICC)	*P*-value	Corrected angle^a^
Wedge angles^b^ (°)	5.2 ± 4.2 (0.82)	2.7 ± 2.4 (0.86)	**<0** **.** **001** *	3.6 ± 3.2
Lordosis angles^c^ (°)	5.1 ± 5.9 (0.86)	7.8 ± 4.6 (0.88)	**0**.014*	2.2 ± 4.9
Axial rotation angles^b^ (°)	3.8 ± 2.6 (0.80)	2.3 ± 2.1 (0.85)	**<0**.001*	3.6 ± 2.5

* *P* < .05. The bold values are *P* values from the paired T test between pre-op and post-op. The bold style is used to underline the significant difference.

^a^ Corrected angles were obtained by a vector calculation of preoperative and postoperative angles.

^b^ For wedge and axial rotation angles, pre-op and post-op were shown as absolute values.

^c^ For lordosis angles, the lordotic angle was defined as negative.

**Figure 2:**
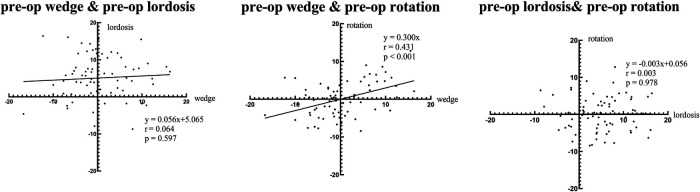
Regression analysis of intervertebral motion before staging surgery.

For intervertebral motions before surgery, there was a strong and significant correlation between the wedge angles and the rotation angles. The lordosis angles showed no associations with either the wedge angles or the rotation angles (Figure 2).

**Figure 3:**
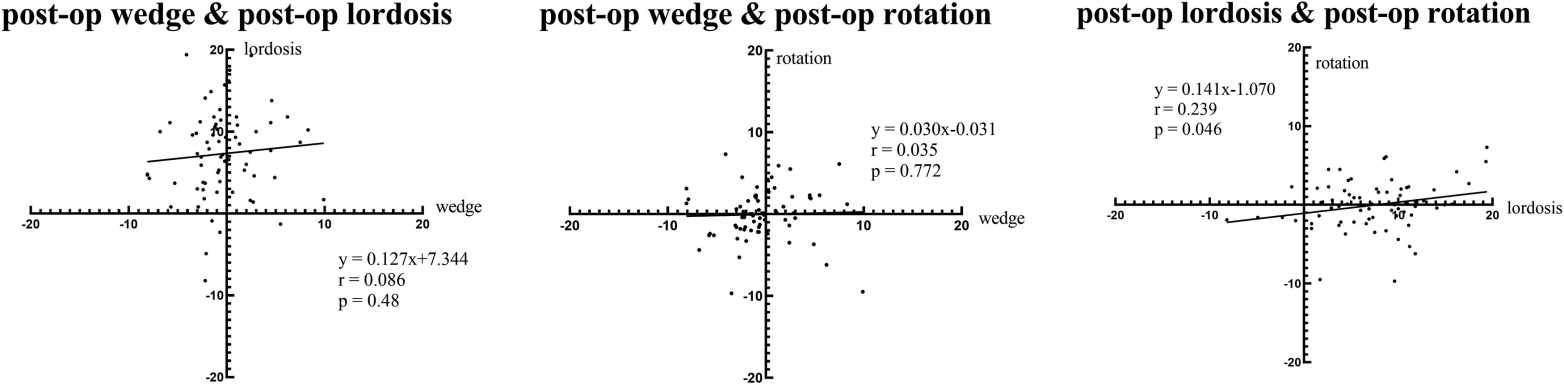
Regression analysis of intervertebral motion after surgery.

For intervertebral motions after first-stage OLIF, a small but significant correlation was found between the lordosis angles and the rotation angles. The wedge angles showed no associations with either the lordosis angles or the rotation angles (Figure 3).

**Figure 4:**
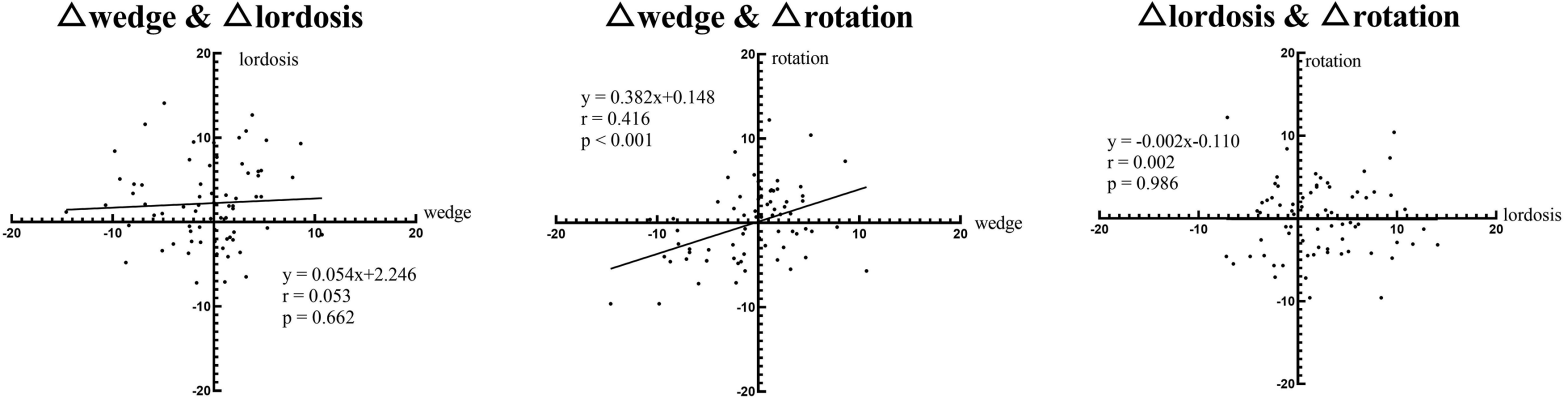
Regression analysis of corrected intervertebral motion within the surgery.

For the corrected intervertebral motions within surgery, further analysis revealed a significant positive correlation between the corrected wedge angles and the corrected rotation angles (Figure 4).

### 3.3. Spinopelvic parameters

Spinopelvic parameters also improved after staging OLIF. LL (27.8° ± 14.9° vs. 33.2° ± 12.5°, *p* < 0.05) increased, while PI-LL (17.9° ± 17.3° vs. 12.7° ± 11.8°, *P* < 0.05), SVA (6.0 cm ± 4.8 cm vs. 2.8 cm ± 3.8 cm, *p* < 0.05), and Cobb angle (15.5 ± 8.2° vs. 7.9 ± 5.6°, *p* < 0.001) decreased. In contrast, there was no difference in TK, PI, SS, and PT before and after surgery (Table 3).

**Table 3:** Spinopelvic parameters and inter-rater reliability before and after surgery.

Spinopelvic parameters	Preoperative	Postoperative	*P*-value	Inter-rater ICC (pre: post)
LL (°)	27.8 ± 14.9	33.2 ± 12.5	0.027*	0.85:0.89
TK (°)	17.4 ± 10.0	19.1 ± 10.8	0.415	0.90:0.91
PI (°)	45.7 ± 14.8	45.8 ± 14.0	0.944	0.82:0.87
PI-LL (°)	17.9 ± 17.3	12.7 ± 11.8	0.041*	0.85:0.84
SS (°)	27.1 ± 10.7	27.1 ± 9.8	0.972	0.88:0.92
PT (°)	18.6 ± 12.5	18.8 ± 11.2	0.920	0.90:0.93
SVA (cm)	6.0 ± 4.8	2.8 ± 3.8	0.001**	0.91:0.90
Cobb angle (°)	15.5 ± 8.2	7.9 ± 5.6	<0.001**	0.86:0.83

* *P* < .05.

** *P* < 0.01.

### 3.4. Representative case

**Figure 5:**
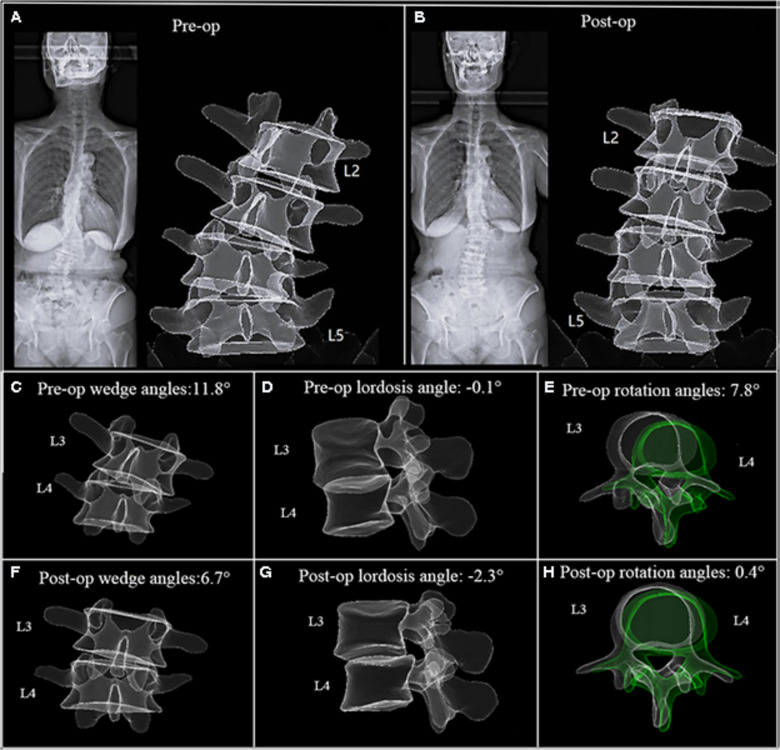
A 69- year-old female patient. (**A**) EOS images and 3D model before surgery. (**B**) EOS images and 3D model 5 days after first-stage OLIF(L2–L5). (**C–H**) The L3–L4 level was selected to demonstrate the intervertebral motion in three planes. The scoliosis was corrected remarkably, corresponding to a reduction in axial rotation.

In the representative case, a 69- year-old female patient presented with numbness in both lower extremities for 2 years (Figure 5), and this condition was diagnosed as adult degenerative scoliosis. OLIF was performed across three levels from L2 to L5. Following the first-stage OLIF surgery, the mean wedge angles decreased from 8.8° to 6.5°, the mean lordosis angle increased from 0.2° to 7.5°, and the mean rotation angles decreased from a preoperative value of 5.9° to a postoperative value of 2.6° (Figures 5A,B). The L3–L4 level was selected to demonstrate the corrective effect 1 week after surgery (Figures 5C–H). The patient reported significant relief in both lower extremities postoperatively with a 2- year follow-up.

## 4. Discussion

This study aimed to investigate three-dimensional intervertebral motions in degenerative lumbar scoliosis and the correlation therein. By re-creating 3D models, we found a strong correlation between the wedge angles and the rotation angles in patients with ADS. In addition, all three intervertebral angles were significantly altered several days after first-stage surgery. However, for the analysis of postoperative data, only a slight correlation was found between lordosis angles and rotation angles, and the coefficient was too small. These findings indicate that the coronal deformity of lumbar vertebrae is associated with intervertebral rotation and loss of lordosis. However, the underlying pathological mechanisms remain poorly understood. Most studies attribute this mechanism to an imbalance of intervertebral load and subsequent degeneration. Ren et al. found ADS patients with vertebral rotatory subluxation inclined to have smaller lumbar lordosis and larger vertebra tilt angles than those without subluxation (8). Bao et al. reasoned that the asymmetry of mechanical stress in the facet joints may contribute to the occurrence of rotatory subluxation, accompanied by a degeneration of the disc and paravertebral muscles (9). Similarly, Ferrero et al. discovered that patients with adult spinal deformity (ASD) with rotatory subluxation exhibited a larger coronal curvature and more severe clinical symptoms (10).

After the first-stage OLIF, a strong positive correlation was observed between the correction amounts in the wedge angles and rotation angles. Since the derotation effect by pedicle screws was not applied in the first stage, this mechanism may be explained by disc excision and cage insertion, which restored the intervertebral height and asymmetry. As a result, the spinal ligamentotaxis by longitudinal ligaments could facilitate a spontaneous 3D reduction efficacy of vertebrae. Moreover, compared with the traditional posterior approach, OLIF minimizes damage to the lamina and paravertebral muscles and facet joints, while maintaining the intrinsic stability of lumbar vertebrae (11). Therefore, first-stage OLIF should be regarded as an effective surgical procedure for correcting both the coronal and the axial deformities across multiple levels with relatively few adverse events.

With respect to spinopelvic parameters, LL and TK increased following the staged surgery, while SVA, PI-LL, and Cobb angle decreased significantly. In the context of sagittal balance theory, the loss of lumbar lordosis is widely acknowledged as the principal pathology of degenerative sagittal imbalance. Compensatory mechanisms to maintain an upright posture and alleviate pain include the retroverted pelvis, reduced thoracic kyphosis, and, in some cases, increased cervical lordosis (12, 13). In our study, the realignment achieved by multilevel surgery can account for the alterations in certain local parameters, while the others may be explained by pain relief and the reversal of spinopelvic compensation. Our findings are consistent with the abovementioned concept, suggesting that OLIF is effective in rectifying sagittal balance and enhancing clinical outcomes.

To our knowledge, this is the first study to investigate the corrective effect of staging OLIF using the EOS image system. Methods for measuring vertebral rotation have evolved significantly over the decades. In the last century, Cobb proposed that rotation could be represented by the spinous process projected in the vertebral cross-section. The Nash–Moe method described that the degree of rotation could be determined by the relative position of the convex pedicle to the vertebral section surface on the frontal x-ray (14). Afterward, Stokes et al. highlighted the importance of considering not only pedicles but also the vertebral shape (width-to-depth ratio) when measuring vertebral rotation, leading to the development of a geometric formula for calculating rotation angles (15). With the rapid advancements in computer science, methodological evolution has shifted toward automation and simulation. Numerous studies have devised automated computerized techniques that demonstrate favorable accuracy and reproducibility in comparison with experienced clinicians (16–18). In our study, we used the EOS image system to measure intervertebral rotations across three planes because of the following advantages: (i) patients are positioned in a functionally loaded standing posture; (ii) all intervertebral rotations are computed automatically ensuring excellent accuracy and reliability (19); and (iii) exposure to low-radiation doses.

We reviewed the previous research focusing on the simultaneous motion effects of adult degenerative vertebrae. Yamaguchi et al. utilized patient-specific 3D CT models to measure the disc height and 3D alignments, including the wedge, lordosis, and axial rotation angles, at each motion segment (20). They found positive correlations between the preoperative wedge and the axial rotation angles, as well as between the corrected wedge and the corrected axial rotation angles, which they referred to as “coupled motion”. However, in their study, patients underwent CT scans in the supine position, while our radiological examinations were performed with the patients in the load-bearing standing position. In addition, the patients in their study underwent lateral lumbar interbody fusion (LLIF), accompanied by posterior stabilization using *in situ* percutaneous pedicle screws (PPS). Rustenburg et al. conducted a biomechanical study noting that an increased degree of scoliosis correlated with more pronounced coupled motions (21). They suggested that the magnitude of coupled motions increased as spinal asymmetry intensified, which supported the coupled motions in the biomechanical area. It should be noted that the concept of “coupled motion” that we used here is not consistent with that of the coupled motion in osteopathy. The latter refers to the accompanying motion in other planes that occurs when the healthy spine actively moves in one plane.

The present study has several limitations. First, it is a retrospective study, with a relatively small sample size. Second, the magnitude of IMAs in three planes in this study is not as significant as in other published studies (22). This discrepancy may be partly due to the IMAs measured here representing the relative rotation of two adjacent vertebrae. Since the deformity of ADS accumulates across multiple vertebrae, it is reasonable that the rotation at each level alone is subtle. Third, 70 surgical levels were not differentiated from L1 to L5. The variables at different levels should be noted. A further study should concentrate on the mechanism and promoting factors of these simultaneous intervertebral motions and their association with clinical outcomes.

## 5. Conclusion

Our study demonstrated a correlation between intervertebral motions in the coronal and axial planes in the natural progression of adult degenerative scoliosis. Moreover, this study also highlighted the effectiveness of OLIF in addressing both rotation and scoliosis concurrently without posterior decompression and instrumentation, as well as improving the sagittal spinopelvic parameters. These findings contribute to a deeper understanding of the underlying mechanisms and potential treatment strategies for adult degenerative scoliosis. However, further research with larger sample sizes and a focus on the mechanisms and contributing factors are warranted to verify and expand upon our findings.

## Data Availability

The raw data supporting the conclusions of this article will be made available by the authors without undue reservation.
